# Cardiovascular Toxicity Induced by Chronic Vincristine Treatment

**DOI:** 10.3389/fphar.2021.692970

**Published:** 2021-07-21

**Authors:** Esperanza Herradón, Cristina González, Antonio González, Jose Antonio Uranga, Visitación López-Miranda

**Affiliations:** ^1^ Departamento de Ciencias Básicas de la Salud, Facultad de Ciencias de la Salud, Universidad Rey Juan Carlos, Alcorcón, Spain; ^2^ Unidad Asociada al Instituto de Química Medica (IQM) del Consejo Superior de Investigaciones Científicas (CSIC), Universidad Rey Juan Carlos, Alcorcón, Spain; ^3^ High Performance Research Group in Experimental Pharmacology (Pharmakom-URJC), URJC, Alcorcón, Spain; ^4^ High Performance Research Group in Physiopathology and Pharmacology of the Digestive System (NeuGut-URJC), URJC, Alcorcón, Spain

**Keywords:** vincristine, cardiovascular toxic effect, sequelae, TNFα, nitric oxide synthase, connexin 43

## Abstract

Vincristine is an effective anticancer agent for treating leukemias, lymphomas, and other solid tumors. Vincristine’s better-known severe side effects include bone marrow depression, hyponatremia, peripheral neuropathy, and gastrointestinal distress. In recent years, cardiovascular damage also has been described during vincristine treatments. However, the vascular toxicity induced by vincristine is little studied. The aim of the present is to evaluate whether these alterations remain after the suspension of chemotherapy treatment (sequelae) and the possible mechanisms involved in this vascular damage. Adult male Wistar rats were used. The animals were divided into four treatment groups: two groups of saline (0.9% NaCl; saline, sequelae saline) and two groups of vincristine (100 μg/kg; vincristine, sequelae vincristine). Saline or vincristine was administered intraperitoneally in two cycles of 5 days each, leaving a rest period between cycles of 2 days. The final cumulative vincristine dose administered was 1 mg/kg. Sequelae groups correspond to 2 weeks after stopping treatment with the antitumor agent. At the end of the different experimental protocols, cardiac and vascular functions were analyzed. Alterations in the expression of different proteins in the cardiovascular tissues were also investigated. Chronic treatment with vincristine did not produce significant changes in basal cardiac function but provoked significant endothelial dysfunction in the aorta and a significant decrease in the mesenteric contractile function. These cardiovascular functional alterations disappeared 2 weeks after the suspension of chemotherapy treatment. Vincristine treatment caused a significant increase in the expression of tumor necrosis factor-alpha (TNFα), endothelial and inducible nitric oxide synthases (eNOS and iNOS), and connexin 43 in cardiac tissue. In the aorta, the chronic treatment with vincristine caused a slight non-significant increase in TNFα expression, a significant increase in eNOS and iNOS, and a significant decrease in connexin 43. After 2 weeks of vincristine treatment (sequelae group), the expression of TNFα increased and eNOS and iNOS expressions disappeared, but a significant decrease in the expression of connexin 43 was still observed in the aorta. In mesenteric arteries, similar data to those found in the aorta were observed. In conclusion, chronic treatment with vincristine causes functional alterations in the vascular function of both conductance and resistance vessels and changes in the expressions of TNFα, eNOS, iNOS, and connexin 43 in cardiovascular tissues, implicating direct toxicity during its treatment. These functional alterations are transitory and disappear after the suspension of its treatment.

## Introduction

Many conventional and newer anticancer agents predispose patients to cardiovascular side effects, including hypertension, acute coronary syndromes, and arterial and venous thrombosis. Therefore, chemotherapy-induced cardiovascular toxicity negatively affects the quality of life and survival of oncological patients, requiring adjustments or discontinuation of the chemotherapy regimen, leading to worse outcomes (Cameron et al., 2016). For that reason, early recognition of cardiovascular dysfunctions associated with the different chemotherapeutic agents becomes imperative (Curigliano et al., 2012; Hamo and Bloom, 2015; Denegri et al., 2016), and understanding cardio-oncology is crucial for effective preventive care (Hong et al., 2010).

Cardiovascular problems are not restricted to anti-angiogenesis drugs alone but are documented with cytotoxic drugs (Schimmel et al., 2004) and the cancer drugs that target microtubules (Sevelda et al., 1994). Among all the possible cardiovascular side effects induced by anticancer agents, cardiotoxicity is the most studied (Madonna, 2017; Bidulescu et al., 2019), but other toxicities and complications can occur with cancer therapies. Vascular toxicity is of particular interest.

Little is known about the possible mechanisms of the vascular complications associated with cancer chemotherapeutics. Vascular toxicity of chemotherapy often reflects endothelial dysfunction, with loss of vasorelaxant effects and suppressed anti-inflammatory and vascular reparative functions. These effects might serve to initiate and further perpetuate the development of hypertension, thrombosis, and atherogenesis (Cameron et al., 2016).

Vincristine, a microtubule-destabilizing drug, is commonly administered as an effective anticancer agent for treating brain tumors, leukemias, lymphomas, and other solid tumors (Murphy et al., 2002; Pui and Evans, 2006; Van Den Bent et al., 2012). Vincristine’s better-known severe side effects that limit clinical utility include bone marrow depression, hyponatremia, peripheral neuropathy, and gastrointestinal distress (Cavaletti and Marmiroli, 2010; van de Velde et al., 2020; Geisler, 2021). However, in recent years, cardiovascular damage also has been described after vincristine treatments. The main cardiovascular side effects provoked by vincristine are myocardial ischemia and infarction, which tend to occur during or shortly after therapy and might therefore be related to coronary artery vasospasm as a result of cellular hypoxia (Meinardi et al., 2000). However, the vascular toxicity induced by vincristine is little studied.

Previously, our research group has described that chronic vincristine treatment causes cardiovascular alterations in rats that mainly affect vascular function, provoking an increase in coronary perfusion pressure and hyperresponsiveness of contractile function and an endothelium-dependent vasorelaxant dysfunction in the aorta (Paniagua et al., 2018). The present study aimed to analyze whether these alterations remain after the chemotherapy treatment suspension (sequelae) and evaluate the possible mechanisms involved in this vascular damage.

## Materials and Methods

### Ethics Statement

Experimental procedures were carried out following the recommendations of the Ethical Committee of the Universidad Rey Juan Carlos (URJC), the Directive 2010/63/EU for the protection of animals used for scientific purposes, and Spanish regulations (RD 109 53/2013).

### Animals

Male Wistar rats (250–300 g; Harlan Iberica, Barcelona, Spain) were used in this study. Animals were maintained in environmentally controlled conditions (adequate cage size; a temperature of 20°C; humidity of 60%) with a 12 h light/12 h dark cycle. Animals were allowed free access to standard laboratory rat chow (Harlan Iberica, Barcelona, Spain) and tap water, which was refreshed every day.

### Treatments

After a week of adaptation to the controlled conditions, we divided the animals into four treatment groups (10–15 animals per group).

Two groups were given saline (0.9% NaCl) or vincristine (100 μg/kg), respectively. Saline or vincristine was administered intraperitoneally in two cycles of 5 days each, leaving a rest period between cycles of 2 days, and the maximum injection volume administered in the animals was 0.5 ml. The final cumulative vincristine dose administered was 1 mg/kg (Aley et al., 1996; Weng et al., 2003).

To determine sequelae of vincristine treatment, two groups, “sequelae saline” and “sequelae vincristine,” were compared. In the sequelae saline group, animals received saline treatment for 10 days, and after 2 weeks of suspension saline treatment, the corresponding experiments were carried out (control sequelae). In the sequelae vincristine group, animals received vincristine treatment, and after 2 weeks of suspension antitumoral agent treatment, the corresponding experiments were carried out. In the sequelae groups, saline and vincristine were administered following the same protocols mentioned above.

### Blood Pressure and Heart Rate Measurements in Anesthetized Rats

In the animals of the different experimental groups, after anesthesia with sodium pentobarbital (50 mg/kg intraperitoneally) (Bencze et al., 2013), direct measurements of systolic (SBP) and diastolic arterial blood pressure (DBP) and heart rate (HR) were carried out based on the method described previously by Herradón et al. (2017). Recording of these cardiovascular parameters lasted for 10 min. After blood pressure measurements, the animals were euthanized and exsanguinated, and the following experiments were performed.

### Isolated Heart Preparation

Basal cardiac function in isolated hearts was evaluated following the methodology described in Herradón et al. (2017). Briefly, hearts were mounted on a Langendorff setup, and coronary perfusion pressure (CPP), left ventricular developed pressure (LVDP), and end-diastolic pressure (EDP) were measured using pressure transducers coupled to a PowerLab 4e recording system (PanLab SA, Barcelona, Spain). These experiments were carried out in 10–15 isolated hearts from each experimental group.

### Aortic Ring Preparations

Vascular reactivity in aorta preparations was performed as previously described (Herradón et al., 2017). Briefly, after cleaned aorta rings were mounted in an organ bath setup, aorta contractile and relaxant reactivity was tested. To assess contractile function, phenylephrine (Phe) (10^−9^ M–10^−5^ M) concentration-response curves were performed. To evaluate vascular endothelium-dependent and endothelium-independent relaxation, carbachol (10^−9^ M–10^−4^ M) or sodium nitroprusside (SNP) (10^−9^ M–10^−6^ M) concentration-response curves were established, respectively, in Phe (1 mM) (submaximal) precontracted preparations. Contraction responses of the aorta rings were expressed as mean absolute values, and relaxation responses are expressed as the percentage of relaxation of the tone induced by Phe.

### Mesenteric Perfused Bed Preparation

Vascular reactivity in the mesenteric perfused bed preparation was evaluated as previously described (Herradón et al., 2017). The experiments were performed in intact mesenteric beds. Mesenteric bed contractile function (Carvalho Leone and Coelho, 2004) was evaluated by a concentration-response curve of Phe (10–80 nmol). The endothelium-dependent vasodilatation was evaluated with a concentration-response curve of carbachol (3 × 10^−10^ mol–3 × 10^−5^ mol), and endothelium-independent vasodilatation with a concentration-response curve of SNP (10^−11^ mol–10^−6^ mol). These experiments were carried out in 10–15 isolated mesenteric beds from each experimental group. Contraction responses of the superior mesenteric artery bed were expressed as mean absolute values, and relaxation responses are expressed as the percentage relaxation of the tone induced by Phe.

### Western Blot Analysis and Tissue Expression of Tumor Necrosis Factor-Alpha, Endothelial Nitric Oxide Synthase, Inducible Nitric Oxide Synthase, and Connexin 43

Different authors have observed that the cardiovascular toxicity of different antitumor drugs (doxorubicin, VEGF-targeting agents, trastuzumab, etc.) modified the cardiac and vascular expressions of TNFα, endothelial nitric oxide synthase (eNOS), inducible nitric oxide synthase (iNOS), and connexin 43. Previous studies by our research group (Herradón et al., 2017) have described that functional alterations after chronic administration of cisplatin at the cardiovascular level are accompanied by structural alterations in cardiac and vascular tissue and changes in connexin 43 and eNOS expression could be related to cardiac functional alterations after cisplatin treatments. For that reason, in the present work, we also evaluated the cardiac and vascular alterations in the expressions of connexin 43, eNOS, and iNOS. On the other hand, a growing body of data indicates that enhanced oxidative stress, inflammation, and upregulation of pro-inflammatory cytokines could be involved in different adverse effects of chemotherapy. For this reason, we also wanted to know if a modification in the expression of TNFα was involved in the cardiovascular functional alterations observed after treatment with vincristine.

For western blot analysis, after different treatments, the heart left ventricle and aorta were dissected and frozen immediately at −80°C. The samples were obtained from 5-6 animals per experimental group.

Protein extraction and total protein values quantification were performed according to the method by (Somoza et al., 2007). After cardiac left ventricle and aorta samples were separated by electrophoresis and transferred onto a PVDF membrane, the membranes were blocked with 3% fat-free milk at room temperature for 1 h and then incubated at 4°C overnight with primary antibody as follows: eNOS (1:500, Cat#610296, BD Transduction Laboratories), connexin 43 (1:6,000, Cat#ab11370, Abcam, Cambridge, United Kingdom), TNFα (1:500, Cat#ab6671, Abcam, Cambridge, United Kingdom), iNOS (1:1,000, Cat#610431, BD Transduction Laboratories), and GAPDH (1:5,000, Cat#ab8245, Abcam, Cambridge, United Kingdom). Afterward, membranes were incubated for 1 h at room temperature with goat anti-mouse (1:10.000, Cat#31430, Thermo Fisher Scientific, United States) or goat anti-rabbit (1:10.000, Cat#31460, Thermo Fisher Scientific, United States) horseradish peroxidase (HRP) conjugated secondary antibody and subsequently treated with Clarity^TM^ Western ECL substrate (Bio-Rad Laboratories). Protein bands were detected using the ChemiDoc XRS + image system (Bio-Rad Laboratories), and the band intensity was assessed with Image Lab software (Bio-Rad Laboratories).

### Histological and Immunohistochemical Analysis

The possible existence of structural damage after treatment with vincristine was analyzed by histological studies following the methodology previously described (Herradón et al., 2017). At the end of the experimental period, samples of heart, aorta, and mesenteric vessels were obtained from the different experimental groups and then fixed and embedded in paraffin. Histological damage was evaluated in sections stained with hematoxylin-eosin stain (HE) and Masson’s trichrome stain. Tissue thickness was measured using the image analysis software package AxioVision 4.6. The analysis was made by triplicate in 5–8 random fields per section and specimen under a 20x or 40x objective. The experimenter was blind to the treatment received by the rat from which the sample under analysis was obtained.

Besides, since methodological problems did not allow us to carry out western blot analysis in the mesenteric bed, immunohistological studies served to deepen the results obtained in the mesenteric bed. Mesenteric samples were processed as previously described (Herradón et al., 2017). Briefly, after heat-induced antigen retrieval in 10 mM citrate buffer for 30 min and blocking endogenous peroxidase, they were incubated overnight at 4°C with the following rabbit polyclonal antibodies: anti-human connexin 43 (1:1,000; Abcam), anti-human tumor necrosis factor-alpha (TNFα; 1:200; Abcam), anti-human eNOS (1:1,000; Novusbio), and anti-human iNOS (1:200; ThermoFisher). After incubation with the primary antibodies, samples were washed, and the peroxidase-based kit ImmPRESS (Vector, MP-7801) was used as a secondary antibody. Samples were then counterstained with hematoxylin and coverslips mounted with Eukitt mounting media (O. Kindler GmbH & Co., Freiburg, Germany). To determine the level of non-specific staining, the preparations were incubated without the primary antibody. ImageJ software was used to calculate the mean intensity of staining in arbitrary units after calculating the maximum threshold value of an average of three images.

### Determinations of the Level of Malondialdehyde

The existence of oxidative damage was determined by levels of MDA in plasma. Levels of plasma MDA were measured by the thiobarbituric acid (TBA) assay (Álvarez et al., 2007). The MDA concentration was measured spectrophotometrically at 535 nm at 20°C, using a microplate reader (FLUOstar Omega, BMG LABTECH). Results were expressed as nmol MDA per ml plasma.

### Compounds and Drugs

Vincristine, phenylephrine, carbachol, and sodium nitroprusside were obtained from Sigma (Sigma Chemical Company, Poole, Dorset, United Kingdom). Phenylephrine, carbachol, and sodium nitroprusside were dissolved in distilled water. Vincristine was dissolved in saline (0.9% NaCl).

### Statistical Analysis

Data are presented as the mean values ± SEM of observations obtained from at least 6 to 15 animals. Statistically significant differences were determined using two-way or one-way ANOVA followed by *post hoc* Bonferroni multiple comparison tests, as appropriate. For western blot analysis, differences between groups were calculated using unpaired Student’s *t*-test, with Welch’s correction where appropriate. Values of *p* < 0.05 were regarded as being significantly different (GraphPad Prism v5).

## Results

### Effects and Sequelae of Vincristine Treatment on Blood Pressure and Heart Rate

Chronic vincristine treatment did not cause any changes in SBP (139.35 ± 10.11 mm Hg, *n* = 13, *p* > 0.05), DBP (104.24 ± 7.71 mm Hg, *n* = 13, *p* > 0.05), or HR (332.20 ± 11.70 beats/minute, *n* = 13, *p* > 0.05) when compared to control group (SPB: 139.52 ± 9.26 mm Hg, *n* = 13; DBP: 104.38 ± 5.80 mm Hg, *n* = 9; HR: 349.87 ± 18.39 beats/minute, *n* = 9).

**FIGURE 1:**
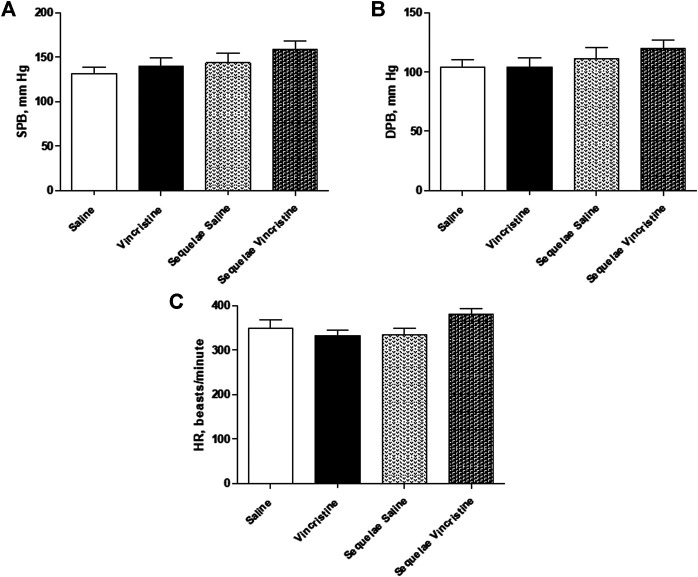
**(A)** Systolic arterial blood pressure (SBP), **(B)** diastolic blood pressure (DBP), and **(C)** heart rate (HR) of anesthetized animals chronically treated with saline (saline treatment) or vincristine (vincristine treatment) and two weeks post-treatment (sequelae saline and sequelae vincristine). Data represent the mean ± SEM, *n* = 6–13 animals per experimental group. A one-way ANOVA followed by Bonferroni’s multiple comparison test was used for statistical analysis.

Two weeks after finishing treatment with vincristine, no changes were observed in blood pressure and heart rate between both experimental groups (vincristine sequelae SBP: 158.25 ± 9.86 mm Hg, *n* = 8; *p* > 0.05 vs. saline sequelae: 143.82 ± 10.47 mm Hg, *n* = 6; vincristine sequelae DBP 120.21 ± 7.09 mm Hg, *n* = 8; *p* > 0.05 vs. saline sequelae: 111.11 ± 9.65 mm Hg, *n* = 6; vincristine sequelae HR: 380.96 ± 11.74 beats/minute, *n* = 8; *p* > 0.05 vs. saline sequelae: 333.75 ± 16.17 beats/minute, *n* = 6) (Figure 1).

### Effects and Sequelae of Vincristine Treatment on Cardiac Function

Chronic vincristine treatment did not cause any significant changes in the left ventricle function (LVDP: 103.76 ± 9.94 mm Hg, *p* > 0.05; EDP: −9.39 ± 5.46 mm Hg, *n* = 11, *p* > 0.05); however, a slight increase in coronary flow (CPP: 104.81 ± 10.46 mm Hg, *n* = 11, *p* > 0.05) was observed when compared to the control group (LVDP: 85.31 ± 5.23 mm Hg; EDP: −4.15 ± 5.71 mm Hg; CPP: 90.58 ± 4.72, *n* = 9).

**FIGURE 2:**
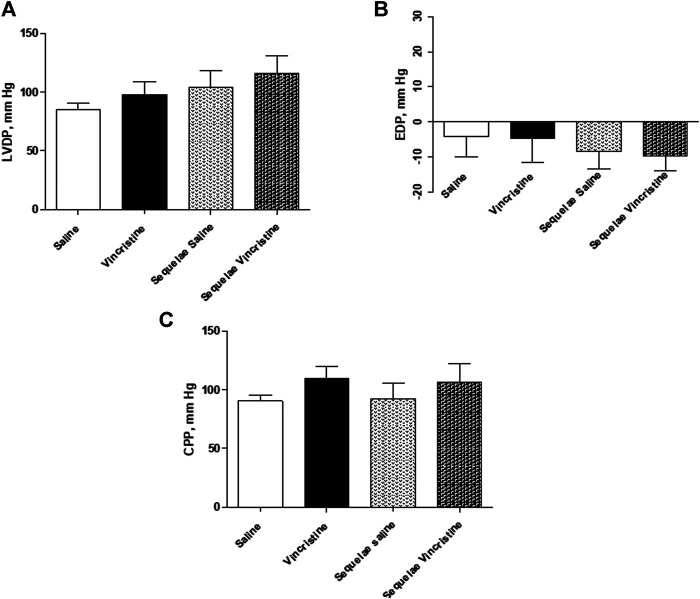
Basal values of **(A)** left ventricular developed pressure (LVDP), **(B)** end-diastolic pressure (EDP), and **(C)** coronary perfusion pressure (CPP) of isolated hearts from animals chronically treated with saline (saline treatment) or vincristine (vincristine treatment) and two weeks post-treatment (sequelae saline and sequelae vincristine). Data represent the mean ± SEM, *n* = 8–12 preparations per experimental group. A one-way ANOVA followed by Bonferroni’s multiple comparison test was used for statistical analysis.

After two weeks of finishing the treatment with vincristine, similar values for left ventricular function or coronary flow were observed (vincristine sequelae LVDP: 116.22 ± 14.82 mm Hg, *n* = 10, *p* > 0.05 *vs.* saline sequelae LVDP: 104.49 ± 14.12 mm Hg, *n* = 8; vincristine sequelae EDP: −9.74 ± 4.15 mm Hg, *n* = 10, *p* > 0.05 vs. saline sequelae EDP: −8.38 ± 4.88 mm Hg, *n* = 8; vincristine sequelae CPP: 106.33 ± 15.55 mm Hg, *n* = 10, *p* > 0.05 vs. saline sequelae CPP: 92.37 ± 13.51 mm Hg, *n* = 8) (Figure 2).

### Effects and Sequelae of Vincristine Treatment on Aortic Vascular Function

**FIGURE 3:**
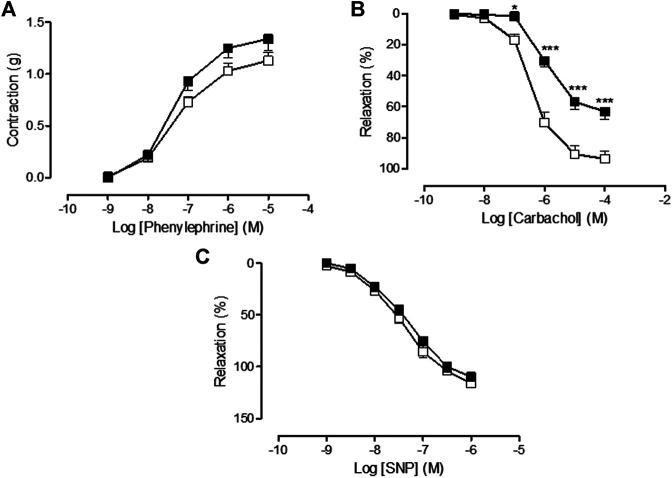
**(A)** Concentration-response curve of phenylephrine (10^−9^ M–10^−5^ M), **(B)** concentration-response curve of carbachol (10^−9^ M–10^−4^ M), and **(C)** concentration-response curve of sodium nitroprusside (SNP) (10^−9^ M–10^−6^ M) in isolated rat aorta rings from animals chronically treated with saline (saline treatment, white squares) or vincristine (vincristine treatment, black squares). Values are expressed as mean ± SEM, *n* = 11–21 preparations per experimental group. A two-way ANOVA followed by the Bonferroni test was used for statistical analysis (***p < 0.001, *p < 0.05, vincristine treatment *vs.* saline treatment).

In the saline-treated group, Phe provoked a concentration-dependent increase in vascular tone resulting in a Rmax value of 1.13 ± 0.08 g (*n* = 15), carbachol caused an endothelial-dependent, concentration-dependent decrease in aortic vascular tone resulting in a Rmax value of 93.69 ± 5.25% (*n* = 10), and SNP caused an endothelial-independent, concentration-dependent decrease in aortic vascular tone resulting in a Rmax value of 116.03 ± 2.69% (n = 13). The treatment with vincristine caused a slight but not significant increase in the aortic vasoconstrictor response to Phe (Rmax = 1.34 ± 0.11 g, *n* = 13, *p* > 0.05 vs. saline) (Figure 3A) but provoked a significant inhibition of the carbachol-dependent endothelium vasorelaxant response (Rmax = 63.18 ± 5.07%, *n* = 21, *p* < 0.01 vs. saline) (Figure 3B). The endothelial-independent vasorelaxation response to SNP was not altered by vincristine treatment (Rmax = 109.74 ± 3.36%, *n* = 11, *p* > 0.05 vs. saline) (Figure 3C).

**FIGURE 4:**
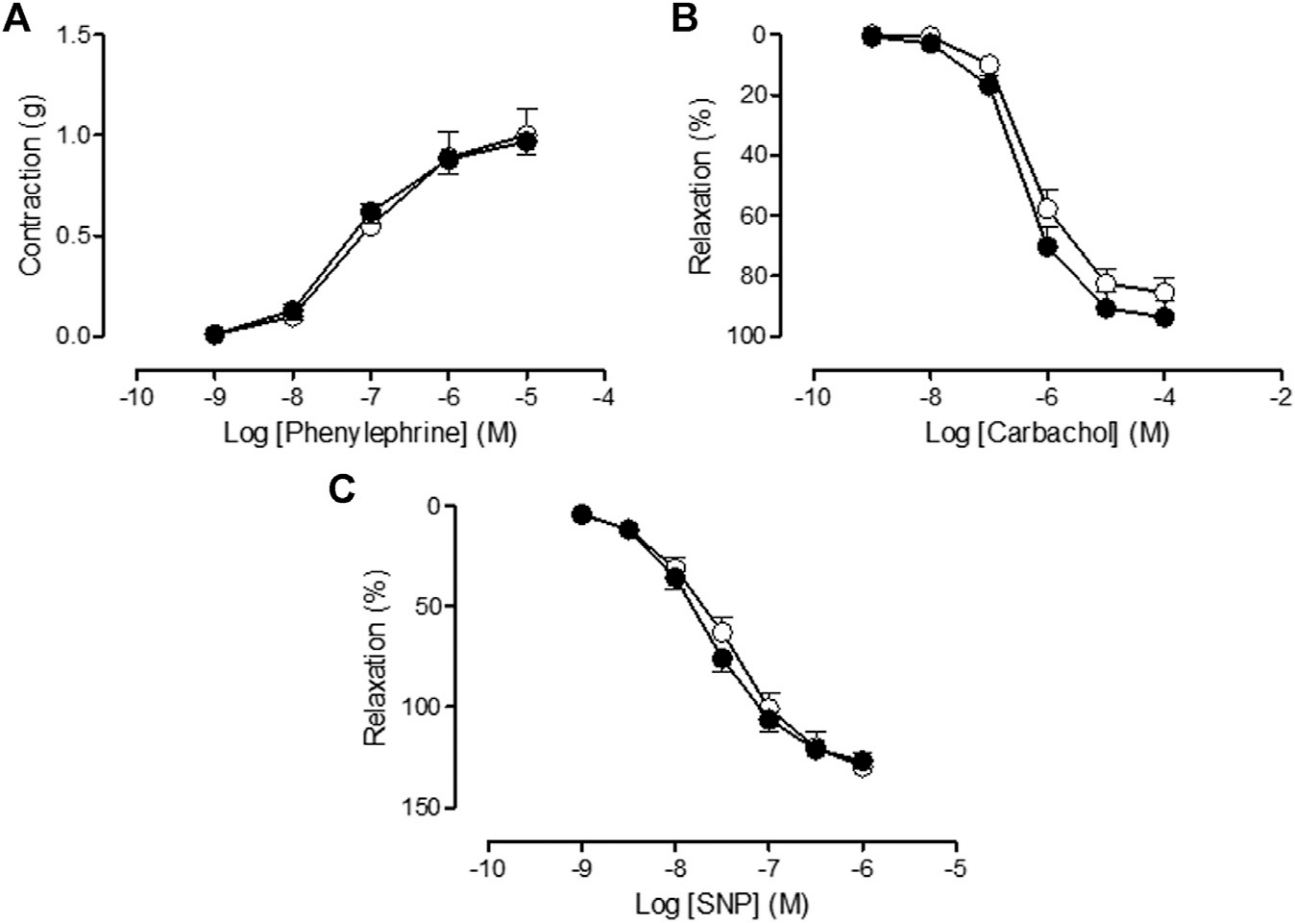
**(A)** Concentration-response curve of phenylephrine (10^−9^ M–10^−5^ M), **(B)** concentration-response curve of carbachol (10^−9^ M–10^−4^ M), and **(C)** concentration-response curve of sodium nitroprusside (SNP) (10^−9^ M–10^−6^ M) in isolated rat aorta rings from animals two weeks post-treatment with saline (sequelae saline, white circles) or vincristine (sequelae vincristine, black circles). Values are expressed as mean ± SEM, *n* = 9–12 preparations per experimental group. A two-way ANOVA followed by the Bonferroni test was used for statistical analysis.

Two weeks posttreatment, similar aortic vasoconstrictor response to Phe (vincristine sequelae Phe: Rmax = 0.97 ± 0.07 g, *n* = 10, *p* > 0.05 vs. saline sequelae Phe: Rmax = 1.00 ± 0.13 g, *n* = 9), endothelial-dependent response to carbachol (vincristine sequelae carbachol: Rmax = 93.69 ± 5.25%, *n* = 10, *p* > 0.05 vs. saline sequelae carbachol: Rmax = 85.50 ± 4.75%, *n* = 12), and endothelial-independent response to SNP (vincristine sequelae SNP: Rmax = 126.68 ± 3.94%, *n* = 9, *p* > 0.05 vs. saline sequelae SNP: Rmax = 129.49 ± 7.24%, *n* = 10) were observed in saline and vincristine sequelae groups (Figures 4A–C, respectively).

### Effects and Sequelae of Vincristine Treatment on Mesenteric Vascular Function

**FIGURE 5:**
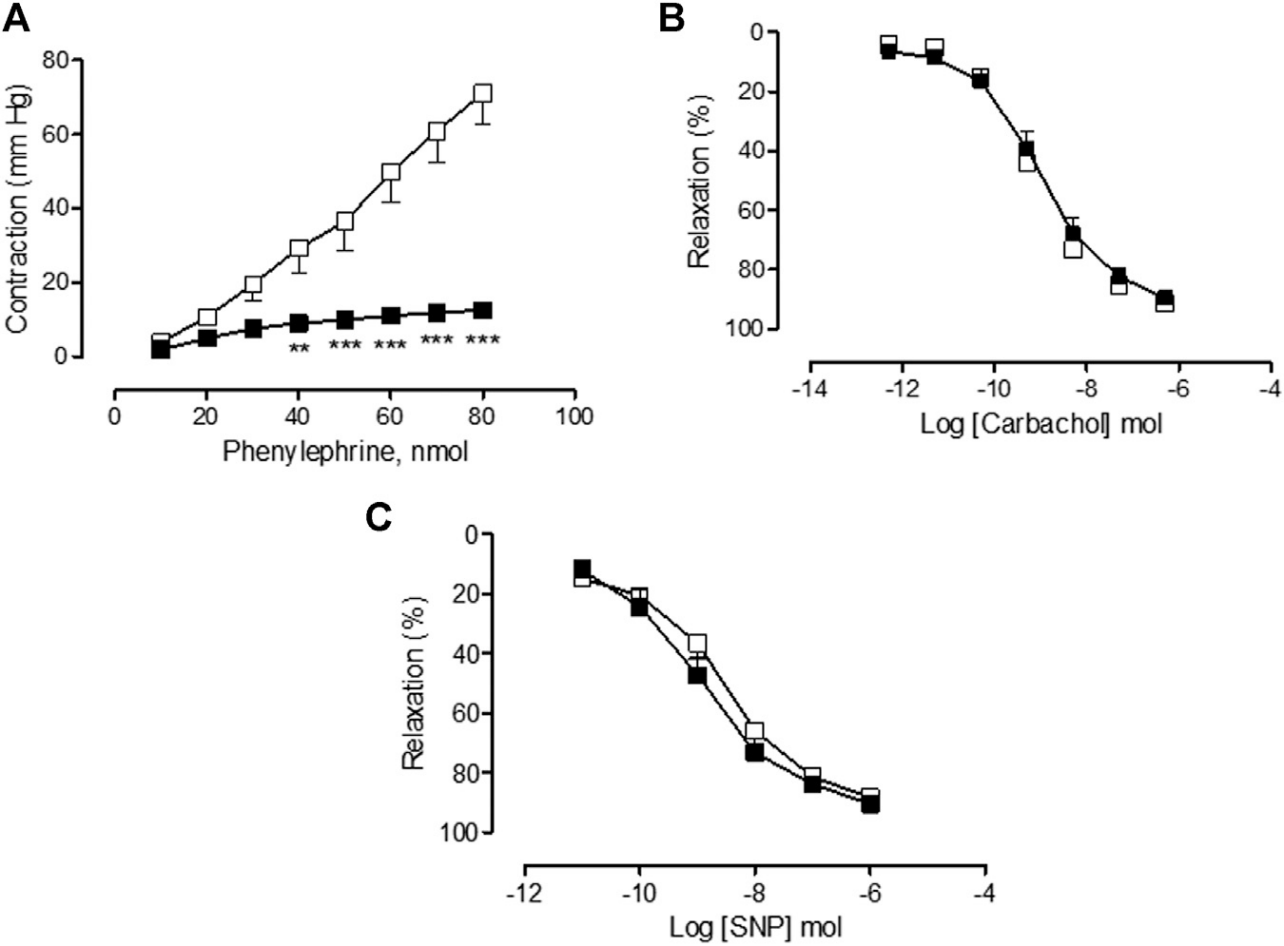
**(A)** Concentration-response curve of phenylephrine (10–80 nmol), **(B)** concentration-response curve of carbachol (3·10^−10^ mol–3·10^−5^ mol), and **(C)** concentration-response curve of sodium nitroprusside (SNP) (3·10^−11^ mol–3·10^−6^ mol) in perfused mesenteric bed from animals chronically treated with saline (saline treatment, white squares) or vincristine (vincristine treatment, black squares). Values are expressed as mean ± SEM, *n* = 9–13 preparations per experimental group. A two-way ANOVA followed by the Bonferroni test was used for statistical analysis (***p < 0.001, **p < 0.01, vincristine treatment *vs.* saline treatment).

In the saline-treated group, Phe provoked a concentration-dependent increase in vascular tone resulting in a Rmax value of 71.06 ± 8.59 1.13 mm Hg (*n* = 9), carbachol caused an endothelial-dependent, concentration-dependent decrease in aortic vascular tone resulting in a Rmax value of 91.14 ± 1.64% (*n* = 9), and SNP caused an endothelial-independent, concentration-dependent decrease in aortic vascular tone resulting in a Rmax value of 88.38 ± 2.74% (*n* = 9). The treatment with vincristine provoked a significant decrease in the mesenteric bed vasoconstrictor response to Phe (Rmax = 12.60 ± 1.81 mm Hg, *n* = 13, *p* < 0.001 vs. saline) (Figure 5A) but did not provoke any modification in the endothelial-dependent (Rmax = 89.54 ± 2.34%, *n* = 8, *p* > 0.05 vs. saline) or endothelial-independent (Rmax = 90.57 ± 1.27%, *n* = 11, *p* > 0.05 vs. saline) vasorelaxant function in the mesenteric bed (Figures 5B,C respectively).

**FIGURE 6:**
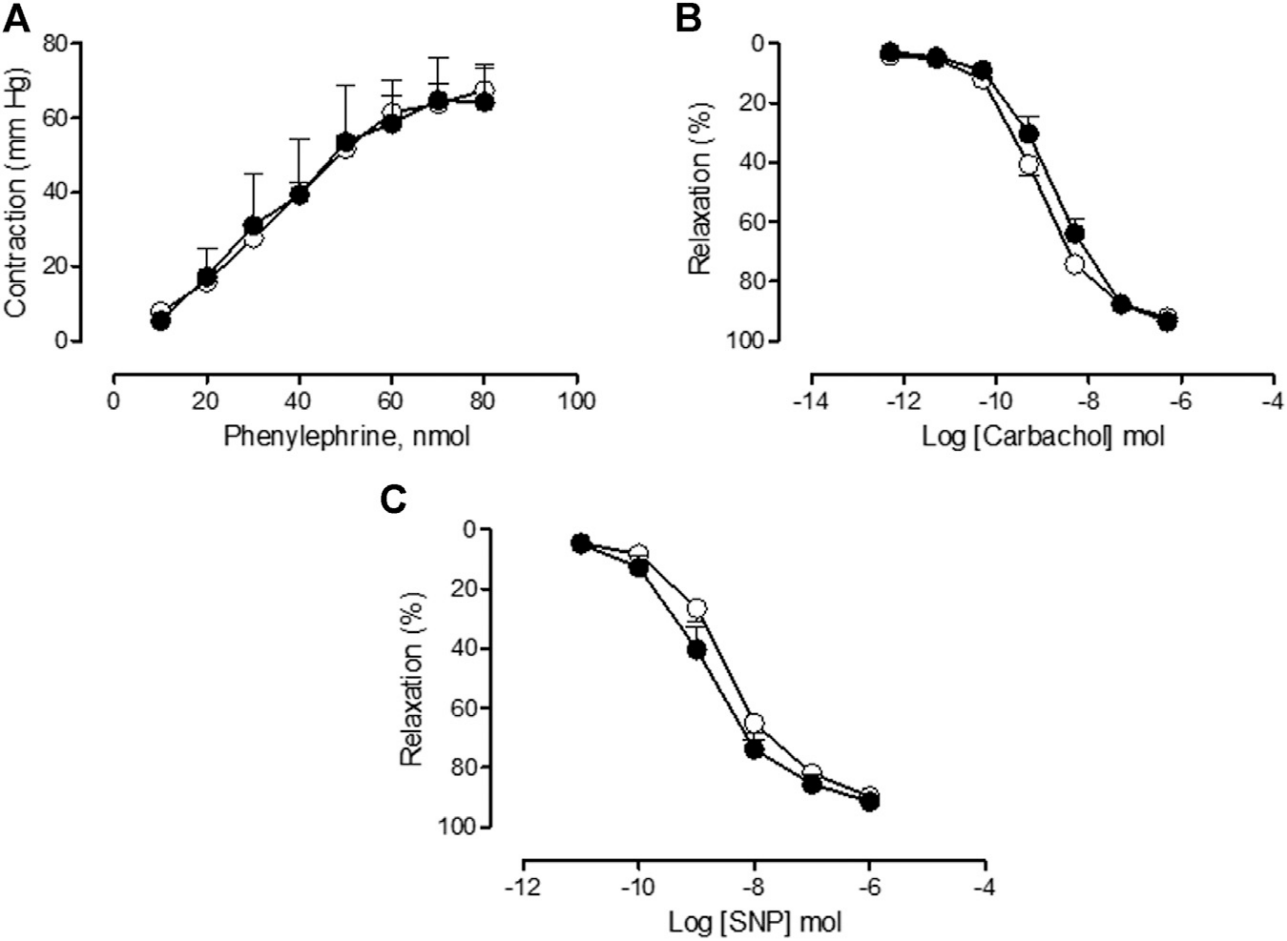
**(A)** Concentration-response curve of phenylephrine (10–80 nmol), **(B)** concentration-response curve of carbachol (3·10^−10^ mol–3·10^−5^ mol), and **(C)** concentration-response curve of sodium nitroprusside (SNP) (3·10^−11^ mol–3·10^−6^ mol) in perfused mesenteric bed from animals two weeks post-treatment with saline (sequelae saline, white circles) or vincristine (sequelae vincristine, black circles). Values are expressed as mean ± SEM, *n* = 6–8 preparations per experimental group. A two-way ANOVA followed by the Bonferroni test was used for statistical analysis.

Two weeks after treatment with vincristine, similar values for mesenteric vasoconstrictor response to Phe (vincristine sequelae Phe: Rmax = 64.23 ± 8.96 mm Hg, *n* = 6, *p* > 0.05 vs. saline sequelae Phe: Rmax = 67.36 ± 6.80 mm Hg, *n* = 7), endothelial-dependent response to carbachol (vincristine sequelae carbachol: Rmax = 93.45 ± 0.68%, *n* = 5, *p* > 0.05 vs. saline sequelae carbachol: Rmax = 92.20 ± 2.36%, *n* = 6), and endothelial-independent response to SNP (vincristine sequelae SNP: Rmax = 91.42 ± 2.14%, *n* = 8, *p* > 0.05 vs. saline sequelae SNP: Rmax = 89.67 ± 2.48%, *n* = 6) were observed in vincristine and saline sequelae experimental groups (Figures 6A–C, respectively).

### Effects Induced by Vincristine Treatment on Expression Cardiac of Tumor Necrosis Factor-Alpha, Endothelial Nitric Oxide Synthase, Inducible Nitric Oxide Synthase, and Connexin 43

**FIGURE 7:**
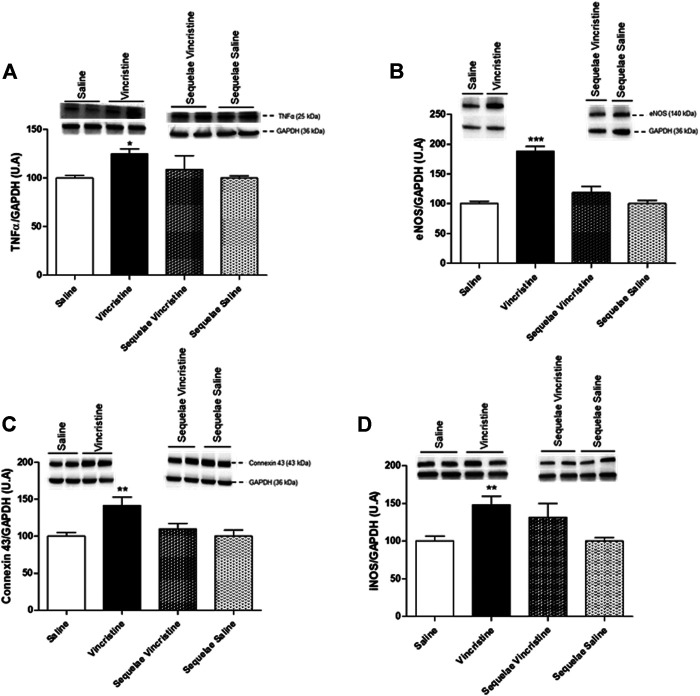
Effect of vincristine treatment and two weeks post-treatment (sequelae) on TNFα **(A)**, eNOS **(B)**, connexin 43 **(C)**, and iNOS **(D)** protein expression in the left ventricle. Representative western blots show bands during vincristine treatment and 2 weeks post-treatment (sequelae). Densitometric evaluations of protein levels were obtained from 4–6 different animals. Data represent the mean ± SEM. Saline *vs.* vincristine (*p < 0.05, *p < 0.05 and ***p < 0.001).

The treatment with vincristine provoked a significant increase in the expression of TNFα (124.67 ± 5.20, *n* = 4, *p* < 0.01 vs. saline: 100.00 ± 2.69, *n* = 5), eNOS (188.09 ± 8.11, *n* = 4, *p* < 0.001 vs. saline: 100.00 ± 4.32, *n* = 6), iNOS (148.04 ± 11.46, *n* = 6, *p* < 0.01 vs. saline: 100.00 ± 6.64, *n* = 6), and connexin 43 (141.41 ± 11.62, *n* = 5, *p* < 0.01 vs. saline: 100.00 ± 5.12, *n* = 6) in the left cardiac ventricle. Two weeks posttreatment with vincristine (sequelae group), the increase in the expressions of TNFα, eNOS, iNOS, and connexin 43 disappeared (TNFα: 108.07 ± 14.20, *n* = 4, *p* > 0.05; eNOS: 118.87 ± 10.16, *n* = 5, *p* > 0.05; iNOS: 131.63 ± 18.23, *n* = 5, *p* > 0.05; connexin 43: 109.81 ± 7.57, *n* = 5, *p* > 0.05), resulting in values similar to those in the saline sequelae group (TNFα: 100.07 ± 2.17, *n* = 6; eNOS: 100.00 ± 5.35, *n* = 6; iNOS: 100.00 ± 4.53, *n* = 6; connexin 43: 100.01 ± 8.17, *n* = 7) (Figure 7).

### Effects Induced by Vincristine Treatment on Aortic Vascular Expression of Tumor Necrosis Factor-Alpha, Endothelial Nitric Oxide Synthase, Inducible Nitric Oxide Synthase, and Connexin 43

**FIGURE 8:**
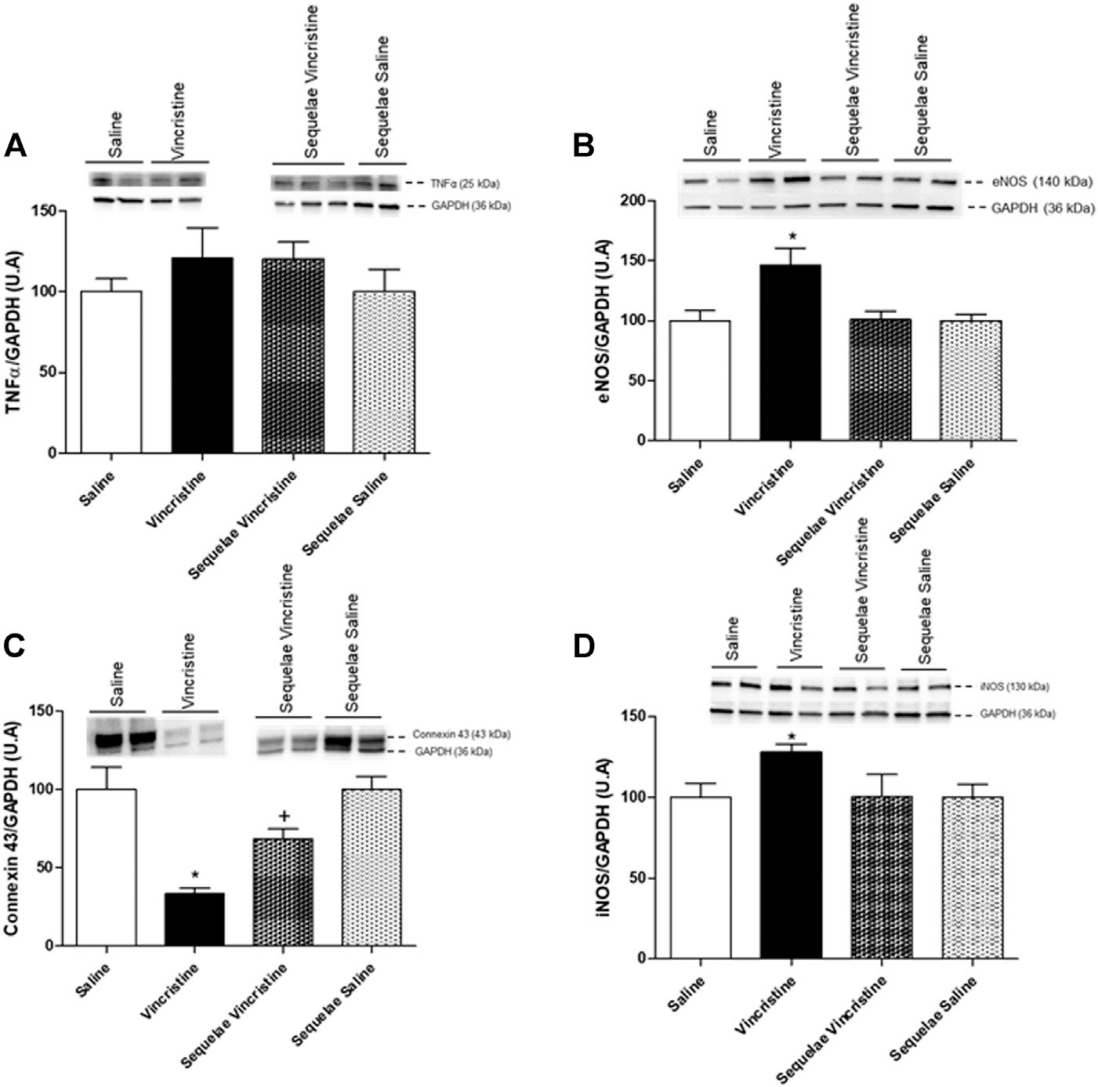
Effects of vincristine treatment and two-week suspension of the treatment (sequelae) on TNFα **(A)**, eNOS **(B)**, connexin 43 **(C)**, and iNOS **(D)** protein expression in the aorta. Representative western blots show bands during vincristine treatment and 2 weeks after treatment (sequelae). Densitometric evaluations of protein levels were obtained from 5–6 different animals. Data represent the mean ± SEM. Saline *vs.* vincristine (*p < 0.05), sequelae saline *vs.* sequelae vincristine (^+^ *p* < 0.05).

The chronic treatment with vincristine did not provoke any modification in the expression of TNFα in aortic tissue (120.82 ± 18.98, *n* = 5, *p* > 0.05 vs. saline 100.00 ± 8.20, *n* = 6). Moreover, no difference was observed in the expression of TNFα 2 weeks after treatment with vincristine (120.68 ± 10.19, *n* = 5, *p* > 0.05 vs. saline sequelae 100.00 ± 13.67, *n* = 6) (Figure 8A).

In the aorta, the expressions of eNOS (146.66 ± 14.04, *n* = 5, *p* < 0.05 vs. saline 100.00 ± 8.79, *n* = 5) and iNOS (128.27 ± 4.48, *n* = 5, *p* < 0.05 *vs.* saline 100.00 ± 8.71, *n* = 6) were significantly increased by the chronic treatment with vincristine. Two weeks after treatment with vincristine (sequelae group), aortic eNOS (101.59 ± 6.91, *n* = 5, *p* > 0.05 *vs.* saline sequelae 100.00 ± 5.32, *n* = 5) and iNOS (100.28 ± 13.90, *n* = 6, *p* > 0.05 *vs.* saline sequelae 100.00 ± 8.19, *n* = 6) expressions were normalized (Figures 8B,D).

Furthermore, the expression of connexin 43 (33.47 ± 3.54, *n* = 6, *p* < 0.05 *vs.* saline 100.00 ± 14.36, *n* = 4) was significantly decreased after chronic treatment with vincristine. Two weeks after treatment with vincristine (sequelae groups), a significant decrease in the expression of connexin 43 (68.40 ± 6.25, *n* = 5, *p* < 0.05 *vs.* saline sequelae 100.00 ± 8.33, *n* = 5) was maintained (Figure 8C).

### Effects Induced by Vincristine Treatment on Mesenteric Vascular Expression of Tumor Necrosis Factor-Alpha, Endothelial Nitric Oxide Synthase, Inducible Nitric Oxide Synthase, and Connexin 43

As previously mentioned, immunohistochemical studies have been carried out in the mesenteric artery to explore in-depth the possible mechanisms involved in the functional alterations found in this tissue.

**FIGURE 9:**
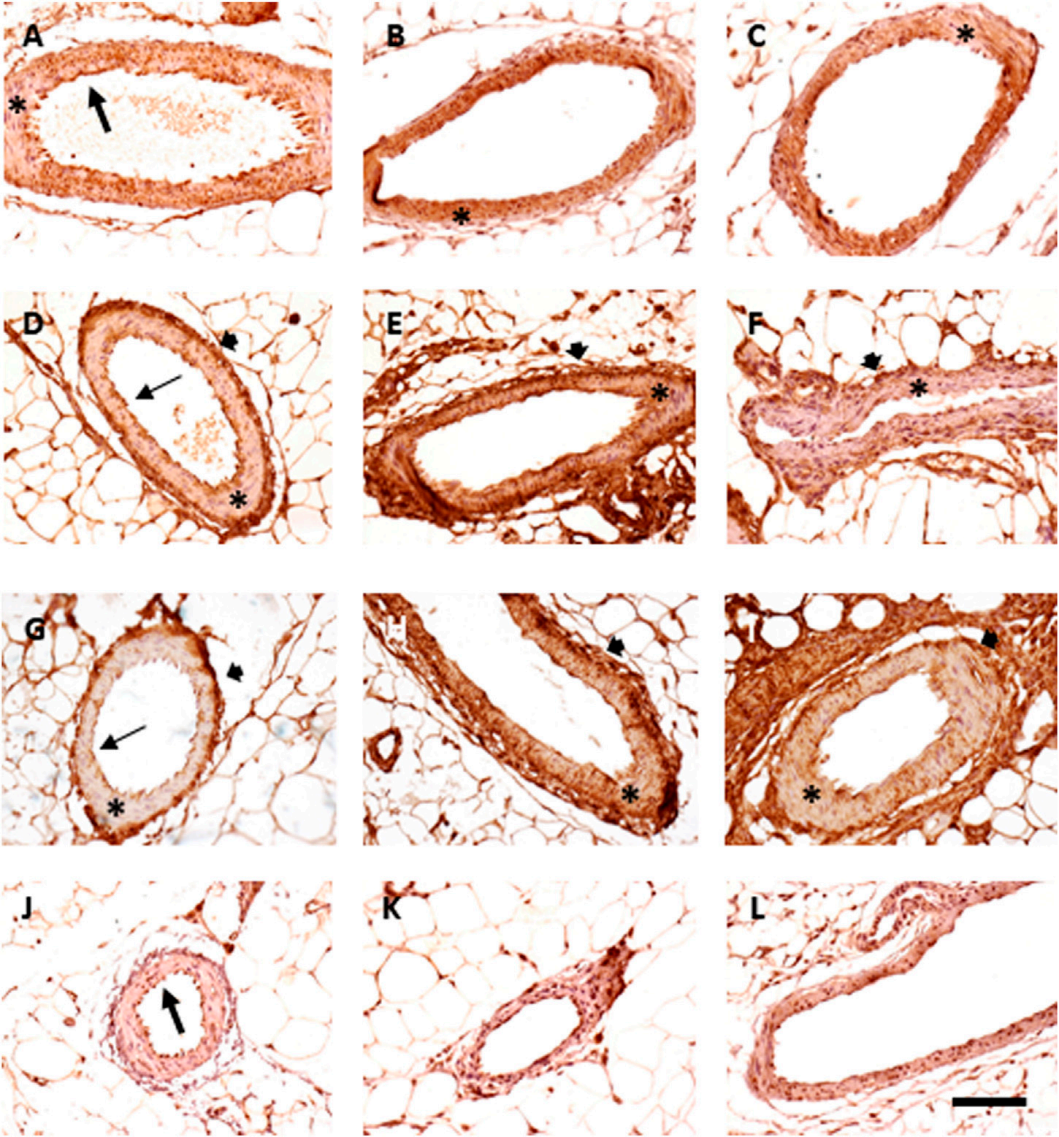
Immunohistochemistry of mesenteric vessels. Expression of TNFα **(A–C)**, eNOS **(D–F)**, and connexin 43 **(G–I)** and iNOS **(J–L)**. Animals treated with saline **(A, D, G, J)**, vincristine **(B, E, H, K)**, and 2 weeks after vincristine administration **(C, F, I, L)**. Bar: 100 μm.

The expression of TNF-α was more intense in the endothelial layer than that in the muscular one, although the chronic treatment with vincristine elicited a certain increase in the staining of the tunica media, which tended to normalize two weeks after treatment since only some positive patches remained. The overall intensity of the staining did not vary between samples. eNOS and iNOS expressions were higher in the endothelial and adventitia layers in vincristine-treated animals than those in the tunica media in animals given saline. Both increased their immunoreactivity in the muscular layer after vincristine treatment though not homogenously (mean intensity of staining increased from 20.7 to 33.8 for eNOS and from 19.4 to 54.2 for iNOS). Two weeks after vincristine treatment, eNOS and iNOS expressions tended to normalize, with intensities of 14.4 and 24.9, respectively. Finally, connexin 43 was mainly negative (mean intensity was 2.5 in saline, 5.4 in vincristine, and 8.6 in sequelae), with some immunoreactivity being restricted to endothelial cells that seemed to be heterogeneously increased with vincristine administration (Figure 9).

### Effects of Vincristine on Cardiac and Vascular Structure

**FIGURE 10:**
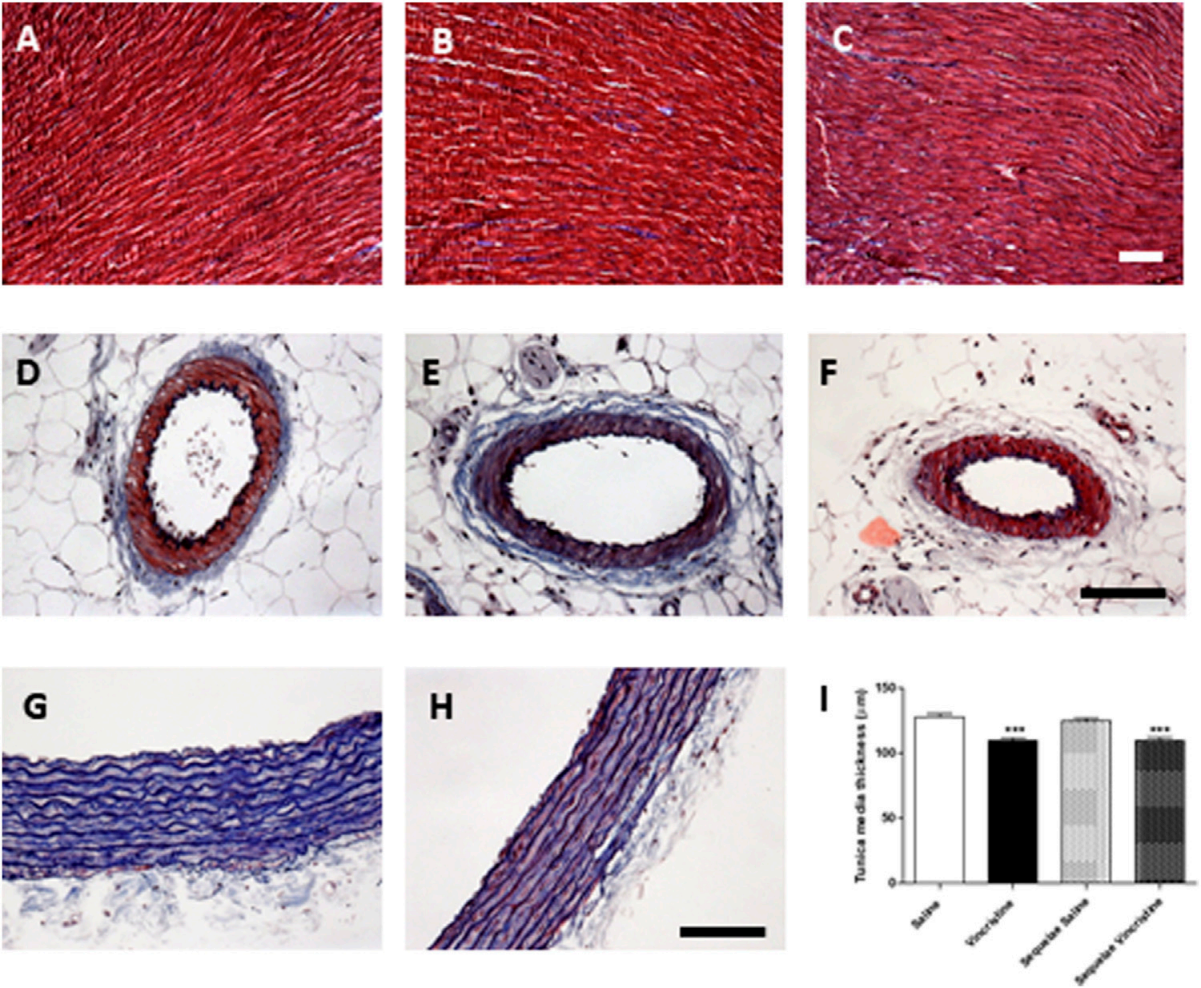
Effect of vincristine in the structure of the heart **(A–C)**, mesenteric vessels **(D–F)**, and aorta **(G–I)**. Masson’s trichrome stain of saline **(A, D, G)**, animals treated with vincristine **(B, E, H)** or vincristine-treated animals 2 weeks after treatment **(C, F)**. Bar: 100 μm. Mean thickness of the aortic tunica media **(I)**, obtained from 4–6 different animals. Data represent the mean ± SEM. Saline *vs.* vincristine (***p < 0.001).

No pathological damage was observed in the histological structure of the heart after vincristine treatment, and no signs of fibrosis appeared in these samples after exposure to vincristine. Similarly, mesenteric arteries did not show changes in the thickness of their muscular lamina after vincristine treatment (mean: 23.8 μm). On the contrary, vincristine treatment significantly reduced the width of the aortic tunica media by 15% (109.8 ± 1.7 μm, *p* < 0.01 *vs.* saline 127.9 ± 2.6 μm). This aortic dystrophy was not recovered two weeks after treatment (sequelae group) (109.5 ± 2.8 μm, *p* < 0.01 *vs.* saline 124.6 ± 1.9 μm) (Figure 10).

### Effects Induced by Vincristine Treatment on Oxidative Damage

**FIGURE 11:**
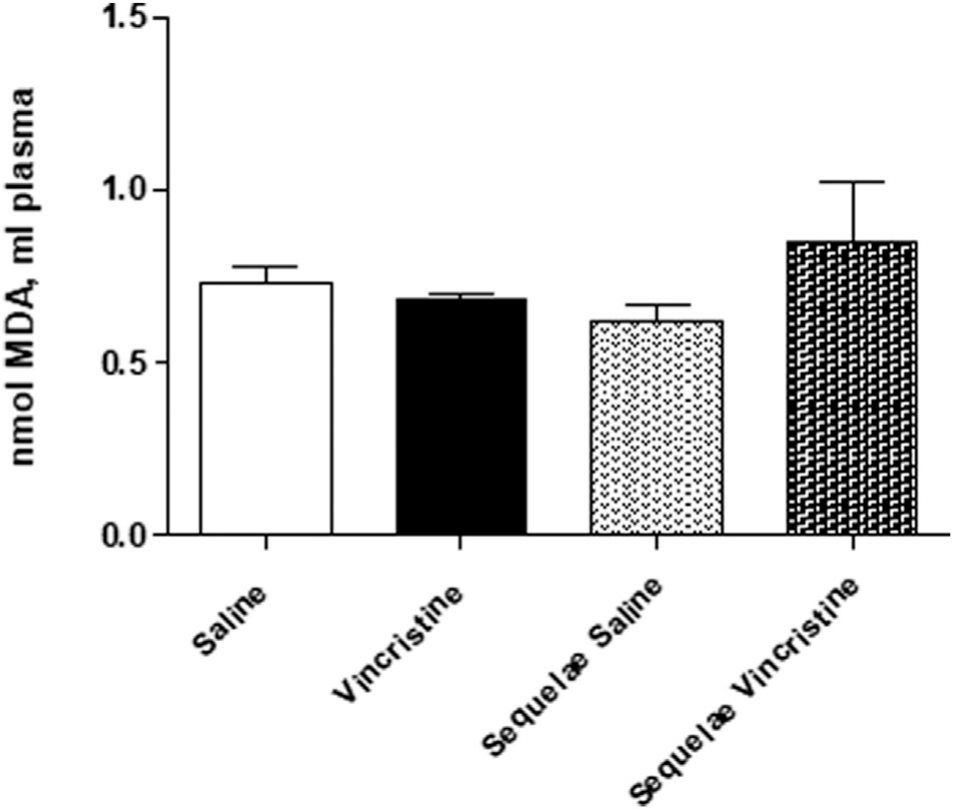
Effect of vincristine treatment and 2-week suspension of treatment (sequelae) on malondialdehyde (MDA) in plasma. Data represent the mean ± SEM, *n* = 4–6 animals per experimental group. A one-way ANOVA followed by Bonferroni’s multiple comparison test was used for statistical analysis.

The concentration of MDA in plasma did not change after vincristine chronic treatment (vincristine: 0.68 ± 0.02 nmol, *n* = 5, *p* > 0.05 *vs.* saline: 0.73 ± 0.05 nmol, *n* = 6) or 2 weeks after treatment with vincristine (sequelae groups) (vincristine sequelae: 0.85 ± 0.17 nmol, *n* = 5, *p* > 0.05 *vs.* saline sequelae: 0.62 ± 0.05 nmol, *n* = 4) (Figure 11).

## Discussion

Through an experimental study, the present work shows that chronic treatment with the antitumor vincristine causes cardiovascular alterations that do not affect all the components of the cardiovascular system similarly. Thus, while alterations are not observed in the general parameters of cardiovascular function such as blood pressure and heart rate or the baseline cardiac function, a significant deterioration is observed in the vascular function, which includes endothelial dysfunction in conductance vessels and contractile dysfunction in resistance vessels. In addition, chronic treatment with vincristine produces alterations in the expressions of TNFα, eNOs, iNOS, and connexin 43 in all the tissues of the cardiovascular system analyzed (heart, resistance vessels, and conductance vessels) and structural alterations in some of them indicate that tissue damage does occur due to the antitumor. After 2 weeks of discontinuation of vincristine treatment, cardiovascular function in the animals and most of the underlying tissue damage were normalized.

Both conventional and new-generation antitumor agents have been reported to predispose patients to cardiovascular complications (Cameron et al., 2016). For this reason, it is increasingly important to establish a balance between the effectiveness of anticancer therapy and the development of the possible risk of cardiovascular complications with the drugs used. In this sense, experimental studies offer a useful alternative for evaluating the toxicity of antitumor agents and their possible mechanisms of action. In this work, the experimental model developed has already been used by different authors for the evaluation of gastrointestinal, hepatic, and hematological toxicity and peripheral neuropathy caused by vincristine (Bessaguet et al., 2018; Paniagua et al., 2018; Geisler, 2021), which validates it as useful for the deeper analysis of the cardiovascular toxicity caused by this antitumor agent. To date, few experimental studies have evaluated the cardiovascular toxicity of vincristine. Previous studies have only demonstrated the presence of cardiac alterations after single doses or short treatments (2 days) of vincristine (Meinardi et al., 2000; Mikaelian et al., 2010; Tochinai et al., 2013), but no studies exist that analyze cardiovascular alterations caused by longer treatments of vincristine and whether these alterations are maintained after the suspension of treatment with the antitumor.

Cardiotoxicity is recognized as a serious side effect of chemotherapy (Bidulescu et al., 2019). Likewise, it has been described that the main cardiovascular side effects of vinca alkaloids, vincristine, and vinblastine are myocardial ischemia and infarction, which tend to occur during or shortly after therapy and might therefore be related to coronary artery vasospasm as a result of cellular hypoxia (Meinardi et al., 2000). The results obtained in the present work show that chronic treatment with vincristine does not produce alterations in the levels of systolic and diastolic blood pressure or the heart rate of the animals. Furthermore, no alterations have been found in either contractile function or left ventricular dilation function during chronic treatment with this antitumor. Other authors have also described similar results since they have demonstrated the lack of association between an alteration in cardiac function and treatment with vincristine (Maraldo et al., 2015). This could suggest the existence of subclinical cardiotoxicity after treatment with this antitumor, in the same way that occurs with other chemotherapeutic drugs such as cisplatin or doxorubicin (Demkow et al., 2011; Sandamali et al., 2020; Xu et al., 2021). It should be noted that, in our study, after chronic treatment with vincristine, we observed a slight, although not significant, elevation in the coronary perfusion pressure that was maintained even after the suspension of treatment. This fact is consistent with that described by Meniardi and the possible development of vasospasm during treatment with vincristine.

As previously discussed, although vascular toxicity caused by antitumor agents has not been widely studied, it is of particular significance since it could be responsible for accelerated atherosclerosis, acute thrombosis, and acute vasospasm in cancer survival patients (Skou Daher and Yeh, 2008; Hedemann et al., 2017). The data obtained in the present work show that after chronic treatment with vincristine, there is significant damage to endothelial-dependent vasodilation in the aorta and severe contractile dysfunction in the mesenteric bed. These findings confirm the results previously obtained by our research group (Paniagua et al., 2018), also demonstrating that these functional alterations at the vascular level appear earlier than others, such as cardiac alterations. Other authors have also shown the presence of endothelial toxicity after treatment with other antitumor drugs such as anthracyclines, cisplatin, taxanes, or 5-fluorouracil (Di Lisi et al., 2017), with this toxicity appearing before cardiac toxicity in the case of cisplatin treatment (Herradón et al., 2017). Regarding the alterations observed in resistance vessels, chronic treatment with vincristine caused a significant decrease in contractile function without affecting vasodilator function. This contractile dysfunction provoked by vincristine could be due to autonomic neuropathy caused by this antitumor (Weissman-Fogel et al., 2008).

Therefore, it can be concluded that vincristine produces significant alterations in vascular function during its treatment that occur before cardiac toxicity, suggesting a greater sensitivity to its toxicity in vascular tissues compared to cardiac cells. Identification of possible endothelial damage caused by antitumor agents can be of great relevance when making decisions about the antitumor treatment to be used to avoid using chemotherapy agents in patients at high risk of vascular complications (Soultati et al., 2012).

To delve into the possible mechanisms by which vincristine causes the functional alterations observed, cardiac and vascular expressions of TNFα, eNOS, and connexin 43 were determined. All these markers have been implicated in the cardiovascular toxicity of other antitumor drugs.

The results obtained in the present study show that treatment with vincristine causes a significant increase in the expressions of TNFα, eNOS, iNOS, and connexin 43 in the cardiac tissue of animals. Similar results have been obtained with other antitumor drugs such as doxorubicin (Lim, 2013; Bin Jardan et al., 2020; Birari et al., 2020), sunitinib (Aldemir et al., 2020), and cisplatin (El-Hawwary and Omar, 2019). On the other hand, enhanced oxidative stress, inflammation, and upregulation of TNFα, interleukin-6 (IL-6), and interleukin-10 (IL-10) have been associated with vincristine-induced neurotoxicity of the peripheral nervous system (Gong et al., 2016; Vashistha et al., 2017; Qin et al., 2020; Geisler, 2021). Furthermore, the increase in these pro-inflammatory cytokines in animals treated with vincristine can be reversed with antioxidant and anti-inflammatory drugs, alleviating the peripheral neuropathy caused by this antitumor (Jiang et al., 2019; Singh et al., 2019). In this way, and although the present study does not analyze whether the development of cardiac alterations with vincristine is mediated by the existence of an underlying inflammatory state, the data obtained in this study are in accordance with this hypothesis.

Different authors have pointed out that cardiac damage produced by vinca alkaloids, such as vincristine, is associated with a deterioration in the cardiac endothelium. Mikaelian et al. have reported, in animals treated by a single dose of vincristine of 1 mg/kg (same dose that has been cumulatively achieved in the present work), an increase in apoptotic markers in myocardial endothelial cells, suggesting that the cardiotoxicity associated with the use of vincristine is due to the damage produced at this level (Mikaelian et al., 2010). Furthermore, acute and chronic cardiotoxicity caused by doxorubicin seems to be due to the generation of hydrogen peroxide by this antitumor agent. On the other hand, it is known that cardiac damage can also result from the increase in peroxynitrite levels from the massive production of nitric oxide by iNOS and its subsequent oxidation. This peroxide caused oxidative damage to cardiac myocytes, thus inducing apoptosis and necrosis (Weinstein et al., 2000; Kalivendi et al., 2001). The results obtained in this study are in accordance with these data since a significant increase in the expressions of eNOS and iNOS was observed. Akolkar et al. have obtained similar results and also demonstrated in isolated cardiomyocytes that treatment with doxorubicin increased the expressions of eNOS and iNOS in this tissue, accompanied by an increase in the generation of TNFα (Akolkar et al., 2017). However, the eNOS and iNOS expression increase without the concomitant existence of functional alterations at the cardiac level could indicate that we are in an early stage of cardiac damage associated with vincristine treatment. However, with doses of vincristine higher than those used in the present work (1.5 mg/kg cumulative dose), other authors have observed degenerative damage and myocardial necrosis after treatment with this antitumor. The type of regimen used may justify the differences in the results obtained. It is known that the toxicity of vincristine depends, among other factors, on the concentration and the duration of exposure (Said and Tsimberidou, 2014).

Many studies have reported that mitochondrial connexin 43 plays an important role in cardioprotection by regulating reactive oxygen special (ROS) signaling and apoptosis, pointing out that, in cardiotoxic situations, the increase in the expression of this protein is an adaptive mechanism to counteract intracellular calcium overload, free radical formation, and propagation of apoptotic signals (Rodríguez-Sinovas et al., 2018). The results obtained in the present study show that connexin 43 levels are significantly increased in cardiac tissue after treatment with vincristine, even before histological tissue damage is observed. These results do not agree with those obtained by other authors in experimental models of doxorubicin-induced cardiotoxicity, in which myocardial histopathological damage has been observed in the form of severe fibrosis accompanied by a decrease in the expression of connexin 43 (Pecoraro et al., 2018; Elhadidy et al., 2020). However, our results agree with those observed in cardiac toxicity caused by trastuzumab (Pecoraro et al., 2020) or cisplatin (Herradón et al., 2017). So, modifications in the expression of connexin 43 and its participation in the cardiac alterations produced by chemotherapy may depend on the drug analyzed.

As already mentioned, to delve into the possible mechanisms involved in vascular alterations observed in vincristine treatment, the vascular expressions of TNFα, eNOS, iNOS, and connexin 43 and the vascular tissue structure were also analyzed. The results obtained in this work show that vincristine causes a slight increase in the expression of TNFα, a significant increase in the expression of eNOs and iNOS, and a significant decrease in the expression of connexin 43 in the aorta. Furthermore, in this tissue, a decrease of around 15% in the thickness of the tunica media is also observed after treatment with vincristine. On the other hand, when analyzing the expression of these markers at the mesenteric tissue level, increases in the expressions of TNFα, eNOS, iNOS, and connexin 43 have been observed, which were not accompanied by histological alterations.

The production of pro-inflammatory cytokines is one of the main risk factors for the development of endothelial dysfunction and a fundamental contributor to the pathogenesis of cardiovascular diseases. It has been demonstrated that TNFα plays a major role in disrupting micro- and macrovascular circulation (McKellar et al., 2009; Zhang et al., 2009). iNOS is activated by external stimuli such as inflammatory cytokines and oxidative stress (Nathan and Xie, 1994). Once iNOS is activated, the nitric oxide produced reacts with the superoxide anion, increasing peroxynitrite, a potent oxidizing agent that has been involved in vascular damage (Beckman and Koppenol, 1996). Furthermore, TNFα in human aortic endothelial cells has been shown to alter the expression of both iNOS and eNOS (Macnaul and Hutchinson, 1993; Villanni F et al., 2008). So, it is possible that the increase in TFNα produced in the vascular tissues by vincristine causes an increase in the expression of iNOS and thus the production of peroxynitrite, which causes the endothelial damage and the hyporesponsiveness observed after treatment with this antitumor in conduit and resistance vascular territories, respectively. Other authors have described similar results in animals treated with doxorubicin, observing an increase in the expression of iNOS in vascular tissue that is accompanied by endothelial dysfunction in this vascular territory (Olukman et al., 2009).

On the other hand, the decrease in nitric oxide (NO) production by the endothelium through eNOS would cause endothelial dysfunction that could lead to cardiovascular disorders. Recent experimental studies have shown that the vascular endothelial growth factor (VEGF) targeting agent, sorafenib, impairs endothelium-dependent vasodilation due to decreased NO levels and the low expression of eNOS (Yu et al., 2021). In addition, it has also been observed that treatment with doxorubicin causes endotheliotoxicity through the production of, among others, ROS at the vascular level and the decrease in the vascular expression of eNOS with the consequent deficit in the production of nitric oxide (He et al., 2019). Our results are not in line with those obtained by these authors since a significant increase in the expression of eNOS and iNOS has been observed both in the aorta and in the mesenteric arteries. It is possible that this increase, in the case of the aorta, is due to a compensatory mechanism for the endothelial dysfunction observed, as has been described in patients with arterial preatherosclerotic changes (Gómez-Fernández et al., 2005). However, this compensatory mechanism would not justify the results obtained in the mesenteric territory where there is a significant increase in the expression of eNOS and iNOS in the absence of endothelial dysfunction. It is also possible that uncoupling of eNOS could also justify the results obtained in this study since this phenomenon is associated with the progression of many pathological conditions such as endothelial dysfunction (Yang et al., 2009). More studies are needed to corroborate this hypothesis in animals treated with vincristine.

Connexin 43 is the main functional component of gap junction in vascular cells. Gap junctional intercellular communication plays an important role in cardiovascular tissue homeostasis. For that, their alterations can be involved in cardiovascular diseases. In the present work, the expression of connexin 43 is significantly decreased in the aorta of animals treated with vincristine, which could also be related to the observed functional alterations. It is known that connexin 43 reduction results in the inhibition of gap junctional intercellular communication activity, increased cell apoptosis, and decreased structural organization and function (Li et al., 2016), which would agree with the results obtained in this work. On the contrary, the expression of connexin 43 is increased in mesenteric arteries, which might not seem like a logical result. However, it has been described that connexin 43 is expressed in a tissue or organ-specific manner to perform a variety of roles (Li et al., 2016). In fact, given that the functional alterations observed in the aorta and mesenteric bed are different, connexin 43 could play a different role in both tissues. In the case of the mesenteric artery, the connection 43 increase could be a compensatory mechanism for the marked hypocontractility observed. This compensatory increase in connexin 43 in situations of hyporesponsiveness has been described in different cardiac pathologies (Michela et al., 2015) or cardiotoxicity induced by doxorubicin (Pecoraro et al., 2015) or trastuzumab (Pecoraro et al., 2020).

Finally, it is also important to note that the decrease in the thickness of the tunica media observed in the aorta after treatment with vincristine could be the consequence of direct damage produced by this antitumor in the aortic tissue. Although this damage has not been appreciated in the case of the mesenteric artery, it could also play a role in the hyporesponsiveness observed at this level. On the other hand, it could also be possible that the decrease in the tunica media of the aorta could be attributed to the decrease in vasodilation induced by the endothelium, which would be manifested in a lower elastic capacity of the arteries. Similar data described by other authors have not been found to corroborate these hypotheses.

On the other hand, it has been described that one of the causes of systemic toxicity caused by antitumor drugs is the production by these agents of ROS, that is, oxidative stress. This oxidative stress could also be the cause of cardiovascular alterations produced by antitumor drugs (Singh et al., 2018). Besides, cellular membranes are vulnerable to oxidation by ROS due to the presence of a high concentration of unsaturated fatty acids in their lipid components, causing lipid peroxidation. The consequences of lipid peroxidation are cross-linking of membrane proteins, change in membrane fluidity, and formation of lipid-protein lipid-DNA adduct, which may be detrimental to the functioning of cells (Conklin, 2004; Winterbourn, 2008). In the present study, whether this lipid peroxidation could be involved in the functional alterations observed at the cardiovascular level was analyzed by evaluating plasma levels of MDA in the animals during treatment with vincristine and two weeks after its suspension. The results obtained in this work show no modifications in this parameter after treatment with this antitumor, thus not demonstrating the implication of this phenomenon in its cardiovascular toxicity. Other authors have shown the development of oxidative stress after treatment with different antitumor agents, including vincristine, and the decrease in toxicity produced after treatment with antioxidants (vitamin A, vitamin E, CN acetylcysteine, etc.) (Singh et al., 2018), although not all results have always been positive, particularly in the case of vincristine. This increase in lipid peroxidation associated with liver toxicity (Harchegani et al., 2019) or peripheral neurotoxicity (Erdoğan et al., 2020) caused by vincristine has been observed in treatments with cumulative doses of vincristine higher than those used in this study. Besides, it is important to mention that a slight but not significant increase in plasma levels of MDA was observed after two weeks of suspension of vincristine treatment. As in other types of injury, there may be an increase in lipid peroxidation after damage caused by vincristine. Other authors have already described that changes in MDA showed different temporal and tissue-specific patterns in models of critical illness. Szczesny and colleagues have described an increase in MDA levels in the heart of animals subjected to burn injury. This increase appears as early as 3 h post-injury and persists even at 40 days post-burn (Szczesny et al., 2015). In any case, more research would be necessary to rule out or confirm the participation of lipid peroxidation in vincristine cardiovascular toxicity since the duration of treatment and the drug regimen used could be not sufficient to produce this lipid peroxidation, which if it occurs, it would probably cause a greater deterioration in the cardiovascular system.

Given the importance of knowing if the cardiovascular toxicity produced by vincristine is maintained or disappears after its treatment suspension, in the present study, an analysis of the parameters evaluated after 2 weeks of suspension of antitumor treatment has been carried out. To our knowledge, this is the first experimental study that analyzes the sequelae of cardiovascular toxicity produced by vincristine. The results obtained show that after 2 weeks of suspension of treatment with vincristine, no changes in the levels of blood pressure and heart rate of the animals or the baseline cardiac function were observed. Moreover, after two weeks of suspension of treatment with vincristine, complete recovery of vascular function both in the aorta and in the mesenteric bed was observed. This indicates that the antitumor vincristine does not produce, in the short term, sequelae in the functioning of the cardiovascular system. The data obtained also show that after suspension of vincristine treatment, cardiac alterations observed in the expressions of TNFα, eNOS, and connexin 43 are not maintained. These data suggest that although the cardiac tissue may suffer from alterations during treatment with vincristine, they are transitory alterations that are not maintained when treatment is suspended. In vascular tissue, the expressions of TNFα, eNOS, and iNOS are completely normalized in the aorta and mesenteric artery after 2 weeks of suspension of treatment with vincristine, maintaining; however, a decrease in the thickness of the tunica media was observed in the case of the aorta. The expression of connexin 43 is completely normalized in the case of the aorta but only partially in the case of the mesenteric bed. These results show that vincristine also causes temporary vascular damage that disappears after its suspension. Although after 2 weeks of suspension of treatment with vincristine, not all the tissue parameters have been recovered at the vascular level, complete normalization may be achieved after a longer period of suspension. Other authors have described the reversible partial effect in cardiac toxicity induced by trastuzumab (Jacquinot et al., 2018) or toxicity induced by doxorubicin (Zhou et al., 2018) after suspension of the treatment. However, the present study is the first that describes this aspect with vincristine.

## Conclusion

In conclusion, the data obtained in this work show that the antitumor vincristine affects the functionality of the cardiovascular system, causing significant impairment in the vasodilator and contractile function in different vascular territories without causing changes in cardiac function. However, this antitumor causes changes in the expression of TNFα, eNOS, iNOS, and connexin 43 in the heart, conductance, and resistance vessels, which could be correlated with direct toxicity in these tissues during its treatment. All these data suggest the need to analyze baseline cardiovascular function in patients before treatment with this antitumor to not aggravate previous pathologies and to monitor cardiovascular function during treatment to control the possible appearance of cardiovascular complications. On the other hand, the results of the present study show that the cardiovascular toxicity caused by vincristine is transitory, disappearing after the suspension of its treatment. Therefore, vincristine could be considered a safe antitumor in terms of the possible short-term and long-term cardiovascular sequelae after its use.

## Data Availability

The original contributions presented in the study are included in the article/supplementary material; further inquiries can be directed to the corresponding author.
